# Diet and the gut-lung axis in cystic fibrosis – direct & indirect links

**DOI:** 10.1080/19490976.2022.2156254

**Published:** 2022-12-27

**Authors:** Isabelle McKay, Josie van Dorst, Tamarah Katz, Michael Doumit, Bernadette Prentice, Louisa Owens, Yvonne Belessis, Sandra Chuang, Adam Jaffe, Torsten Thomas, Michael Coffey, Chee Y. Ooi

**Affiliations:** a School of Clinical Medicine, Discipline of Paediatrics and Child Health, UNSW Medicine and Health, Univeristy of New South Wales, Randwick, Australia; b Department of Nutrition and Dietetics, Sydney Children’s Hospital Randwick, Randwick, Australia; c Department of Physiotherapy, Sydney Children’s Hospital Randwick, Randwick, Australia; d Molecular and Integrative Cystic Fibrosis (miCF) Research Centre, University of New South Wales, Randwick, Australia; e Department of Respiratory Medicine, Sydney Childrens Hospital, Randwick, Australia; f Biological, Earth and Environmental Sciences, University of New South Wales, Randwick, Australia; g University of New South Wales, Centre for Marine Science and Innovation, Randwick, Australia; h Department of Gastroenterology, Sydney Children’s Hospital, Randwick, Australia

**Keywords:** Nutrition, microbiome, cystic fibrosis, gut-lung axis, *akkermansia*

## Abstract

Cystic fibrosis (CF) is a multisystem, autosomal, recessive disease primarily affecting the lungs, pancreas, gastrointestinal tract, and liver. Whilst there is increasing evidence of a microbial ‘gut-lung axis’ in chronic respiratory conditions, there has been limited analysis of such a concept in CF. We performed a comprehensive dietary and microbiota analysis to explore the interactions between diet, gastrointestinal microbiota, respiratory microbiota, and clinical outcomes in children with CF. Our results demonstrate significant alterations in intestinal inflammation and respiratory and gastrointestinal microbiota when compared to age and gender matched children without CF. We identified correlations between the gastrointestinal and respiratory microbiota, lung function, CF pulmonary exacerbations and anthropometrics, supporting the concept of an altered gut-lung axis in children with CF. We also identified significant differences in dietary quality with CF children consuming greater relative proportions of total, saturated and trans fats, and less relative proportions of carbohydrates, wholegrains, fiber, insoluble fiber, starch, and resistant starch. Our findings position the CF diet as a potential modulator in gastrointestinal inflammation and the proposed gut-lung axial relationship in CF. The dietary intake of wholegrains, fiber and resistant starch may be protective against intestinal inflammation and should be explored as potential therapeutic adjuvants for children with CF.

## Introduction

Cystic fibrosis (CF) is a multisystem autosomal recessive disease, with a high morbidity and mortality burden.^1^ Advances in nutritional and pulmonary therapies over the last few decades have modified the natural history of CF, and we have now reached a point where the life expectancy in CF has increased such that over half the individuals living with CF in countries like Australia (53.7%) and the United States (56%) are adults.^1,2^ Adult-specific gastrointestinal complications, including cancer, are now emerging concerns.^3^

One of the key clinical principles underpinning this survival success was the implementation of a high-fat, high-energy diet to counteract the increased energy demands (through malabsorption and respiratory disease) which predominates in CF.^4^ However, this dietary regimen has remained relatively unchanged since its introduction four decades ago, and as the CF population continues to live longer into adulthood, the longer-term effects of this modified diet may require reevaluation. A contemporary analysis of the CF diet revealed that these energy requirements are commonly being met through the consumption of ‘junk foods’.^5^ There is also evidence demonstrating that these energy-dense nutrient-poor foods may have correlations with pro-inflammatory conditions such as metabolic disease and gastrointestinal cancer.^3,5–8^

It has long been established that altered gastrointestinal microbial profiles and decreased microbial diversity (dysbiosis) are associated with disruptions to homeostasis and disease progression.^9,10^ Marked respiratory and gastrointestinal dysbiosis,^11–13^ in conjunction with significant gastrointestinal inflammation,^11,14–16^ have now become recognized as hallmarks of the CF milieu. There is an increasing body of evidence supporting a microbial ‘gut-lung axis’ in chronic respiratory conditions such as asthma and chronic obstructive pulmonary disease, impacting upon common mucosal immunity across these organ systems.^17–20^ However, evidence of this relationship in the unique multisystem context of CF is scarce.^21^

While diet is a known modulator of gastrointestinal microbial communities,^22^ there is a paucity of research investigating the relationship between the high-fat CF diet and the gastrointestinal and respiratory microbiota and markers of disease/health outcomes in CF. Madan and colleagues^23^ described a relationship between infant breastfeeding and respiratory microbial diversity in CF, as well as a correlation between solid foods and gastrointestinal biodiversity. Hoen et al.^24^ illustrated a similar premise, specifically time to first CF exacerbation and the relationship with diet and microbial health. Some dietary components – including resistant starch and dietary fiber – have been described as beneficial for metabolic health in the general population but remain largely unexplored in CF.^25–28^ Further, there is little evidence on the potential mechanisms underlying microbial immune modulation and the gut-lung axis in CF.^21^

The aims of this study were to: 1) characterize differences in dietary intake, gastrointestinal and respiratory microbiota, and biomarkers of disease between CF participants and healthy controls (HC); and 2) explore any potential associations within the CF group between i) diet, ii) gastrointestinal microbiota, iii) respiratory microbiota, iv) gastrointestinal inflammation and v) clinical outcomes.

## Materials and methods

### Study design

This was a prospective, cross-sectional, controlled observational study, comparing a cohort of children with CF to a cohort of HCs, free of any chronic disease. The study forms part of the EARTH (**E**valuating the **A**limentary and **R**espiratory **T**racts in **H**ealth and disease) program (ethical approval HREC/18/SCHN/26), described in detail in the previously published protocol.^29^ The study was a single-center study conducted at an Australian tertiary pediatric hospital – the Sydney Children’s Hospital (SCH) in Randwick, Australia – from April 2018 to September 2019. Inclusion criteria were as follows: i) aged between 0 and 18 years; ii) diagnosed with CF according to the Cystic Fibrosis Foundation consensus criteria^30^ (CF group) or free of any chronic health condition (HC group); and iii) provided informed consent if 16 years of age or older or a parent(s)/carer(s) provided informed consent on their behalf. Exclusion criteria included: i) children with more than one concurrent or unrelated chronic disease; ii) children with CF currently on CFTR modulators; iii) inability to comply with study requirements; and iv) participant/guardian inability to speak English or a reading level lower than 12 years of age.

### Sample collection and analysis

All subjects were requested to provide a stool and an airway (sputum or oropharyngeal swab if non-productive) sample, collected as per the EARTH program protocol.^29^ Stool and airway samples underwent DNA extraction and 16S ribosomal RNA gene sequencing. DNA was extracted using the QIAamp Fast DNA Stool Mini Kit and QIAamp DNA Mini Kit (QIAGEN, Hilden, Germany) for stool and airway samples, respectively, according to the manufacturer’s instructions. Community 16S rRNA genes were amplified with the primers 515 F (GTGYCAGCMGCCGCGGTAA) and 806 R (GGACTACNVGGGTWTCTAAT) spanning the V4 region. 16S rRNA sequencing was performed using the Illumina MiSeq platform at the Ramaciotti Center for Genomics (University of New South Wales, Sydney, Australia). Quality filtering was performed according to the thresholds outlined in Coffey et al. 2019.^31^ Processed sequences were clustered in unique sequences (zero-distance operational taxonomic unit; zOTU) with the unoise2 algorithm implemented in USEARCH. After chimera removal, sequences were then classified by BLASTn alignment against the SILVA database. Concatenated sequences of all sequences were mapped on the final set of zOTUs to calculate the abundance of each zOTU for each sample. Stool calprotectin (as biomarker of gastrointestinal inflammation) was measured from the stool sample using a monoclonal enzyme-linked immunosorbent assay (EK-CAL Calprotectin ELISA, Bühlmann, Switzerland), according to the manufacturer’s instructions. The upper limit of linearity for the assay was 600 µg/g; samples giving results above this level were subject to further dilutions to provide a quantitative result.

### Dietary surveys and analysis

Dietary intakes were quantified by the Australian Child and Adolescent Eating Survey (ACAES), a 120-item semi-quantitative food frequency questionnaire measuring intakes over the preceding 6 months (validated for Australian participants aged 2 years or older) (University of Newcastle, Australia)^32^ according to the methods outlined in the EARTH protocol.^29^ Children under 2 years of age underwent a dietitian-administered 24-hour food recall, measuring intakes over the preceding day. Nutrient intake data from the recalls was extracted using FoodWorks (v9) and the following databases: AusFoods 2017 and AusBrands 2017 (Xyris Software, Australia). A registered dietitian inspected the recalls for completeness and plausibility.

### Clinical surveys and analysis

Each participant was asked to complete a clinical questionnaire for the collection of demographic, and anthropometric information. This questionnaire was designed and distributed through Qualtrics (Qualtrics, Provo, Utah, USA).^33^ Clinical data for CF patients were obtained through medical chart review (clinician confirmed), and included genotype, exocrine pancreatic status, *Pseudomonas aeruginosa* status, frequency of CF pulmonary exacerbations (CFPE) and pulmonary function (spirometry). Pulmonary function tests were limited to individuals >5 years old and measured as per ATS/ERS guidelines using the reference Global Lung Index values.^34,35^

### Statistical methods

Statistical analyses were performed in RStudio (v3.6.0).^36^ Categorical variables were compared using Fisher’s Exact Test for count data; continuous variables were analyzed according to distribution with a Student’s t-test or Mann-Whitney U test for parametric and non-parametric data, respectively. Generalized linear models were used to control for age when comparing continuous variables between groups. Alpha diversity was assessed by richness (number of zOTUs) and the Shannon-Weaver index. Beta diversity was calculated using relative abundance and presence/absence data and Bray-Curtis dissimilarity to generate non-metric multidimensional scaling (NMDS) plots. Permutational multivariate analysis of variance (PERMANOVA) tests were conducted using the *adonis* function (*vegan* package) and determined whether beta diversity was significantly different between cohorts.^37^ Significant differences in taxa abundances between cohorts were determined using the ANCOM package v1.1–3 and corrected for multiple testing (false discovery rate (FDR) <0.05).^38^ Correlations between two continuous variables were performed using Spearman correlations (adjusted p-values, denoted by ‘q’, were produced using Benjamini and Hocheberg correction for multiple testing). Graphs were produced using the *ggplot2* package.^39^

## Results

### Participants

Eighty-two participants meeting the eligibility criteria were recruited: 41 in the CF population, and 41 in the HC population (Table 1). Of these, 19 CF (all pancreatic insufficient) and 19 HC completed all elements of the study, including stool/airway samples, a clinical survey, and a food frequency questionnaire. All 82 participants were divided into four subset groups (based on which study elements each participant fully completed): airway analysis, stool analysis, airway-stool analysis and diet analysis (Table 1). All study groups were matched for sex and age, except for the dietary analysis group, which, based on spread of completed ACAES surveys, was age-matched only. All datasets generated and analyzed in the current study are available in the figshare repository, DOI: 10.6084/m9.figshare.19416830.

**Table 1.** Demographics and descriptive statistics for study participants.

	CF	HC
***Airway analysis (n = 82)***	**41**	**41**
Age (years, median [IQR])	9.72 [5.7, 13.5]	10.08 [5.3, 14.3]
Female, n (%)	20 (48.8)	20 (48.8)
HAZ (SD)	0.11 (1.02)	−0.52 (1.2)
WAZ (SD)	0.21 (0.82)	0.42 (1.25)
BMIZ (SD)	0.41 (0.72)	−0.004 (1.01)
Sample type: swab	29	41
Sputum	12	0
Pancreatic Insufficient/Sufficient	36/5	n.a.
Genotype: F508del homozygous F508del heterozygous Other	17 18 6	n.a.
***Stool analysis (n = 66)***	**33**	**33**
Age (years, median [IQR])	9.58 [5.28, 2.51]	8.87 [4.91, 13.8]
Female, n (%)	17 (51.5)	17 (51.5)
Pancreatic Insufficient/Sufficient	29/4	n.a.
Genotype F508del homozygous F508del heterozygous Other	17 12 4	n.a.
***Airway-stool analysis (n = 60)***	**30**	**30**
Age (years, median [IQR])	9.43 [4.4, 11.6]	8.78 [5.1, 13.9]
Female, n (%)	17 (56.6)	17 (56.6)
Pancreatic Insufficient/Sufficient	27/3	n.a.
Genotype F508del homozygous F508del heterozygous Other	15 12 3	n.a.
***Dietary analysis (n = 38)***	**19**	**19**
Age (years, median [IQR])	9.30 [5.5, 12.1]	9.20 [5.8, 12.5]
Female, n (%)	10 (52.6)	10 (52.6)
Pancreatic Insufficient/Sufficient	18/1	n.a.
Genotype F508del homozygous F508del heterozygous Other	13 5 1	n.a.

WAZ = average weight z scores, HAZ = average height z scores, BMIZ = average body mass index z scores, SD = standard deviation, IQR = interquartile range.

WAZ = average weight z scores, HAZ = average height z scores, BMIZ = average body mass index z scores, FEV_1_ = average forced expiratory volume in 1 second, FEV_1_% pred = average predicted FEV_1_ based on reference Global Lung Index (GLI) values, FVC = average forced vital capacity, FVC = average predicted FVC based on GLI values. CFPE = Cystic Fibrosis pulmonary exacerbactions, (mean) = average number of *Pseudomonas aeruginosa* infections, detected pathogens of interest and CFPE in the last 3 years for patients with CF (All), (patients 1–10 yrs), or (patients >10 years old).

### Comparisons between participants with cystic fibrosis and healthy controls

#### Altered diet in cystic fibrosis

**Figure 1.**
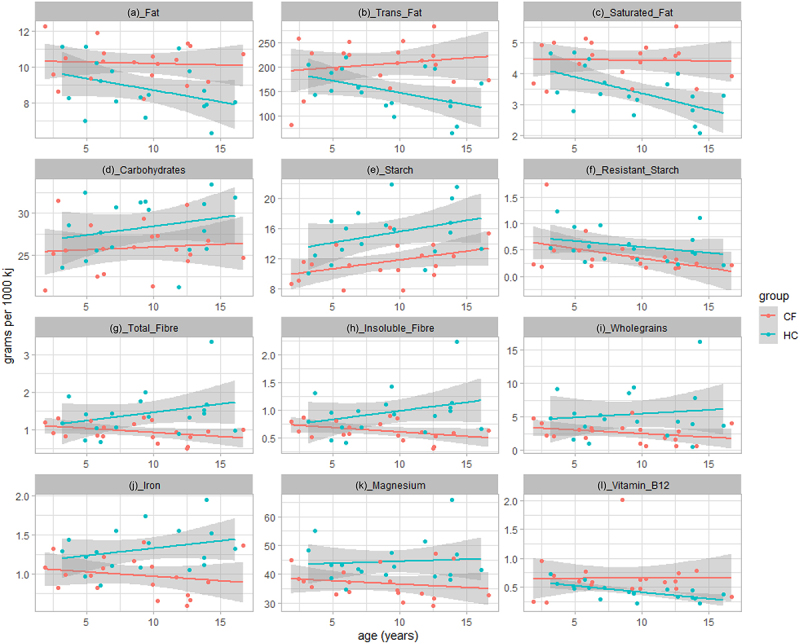
Significant differences in macro- and micronutrient intake between CF (n = 19) (red) and HC (n = 19) (blue) groups. Values are adjusted for total energy intake. Shaded regions represent 95% confidence intervals constructed from generalized linear models controlling for age; solid line represents mean.

In line with the CF recommended dietary guidelines, results from the ACAES survey demonstrated that children with CF had a significantly higher energy intake (median [IQR] of 11531 kilojoules (kJ)/day [6856–14376]) compared to the HC group (median 7331 kJ/day [5788–9067]) (*p* = .008) (Supplementary data Table 1). To better understand diet quality, we evaluated diet as relative intake (per/1000 kj). Once adjusted, CF participants had a reduced relative intake of carbohydrates (mean difference of −2.6 g, *p* = .03), starch (mean difference = −4.6 g, *p* = .004), resistant starch (mean difference = −0.3 g, *p* = .007), wholegrains (mean difference of −1.7 g, *p* = .006), total fiber (mean difference of −0.5 g, *p* = .001), insoluble fiber (mean difference of −0.3 g, *p* = .001), and an increased relative intake of total fats (mean difference of 1.9 g, *p* = .003), saturated fats (mean difference of 1.3 g, *p* = .001), and trans-unsaturated fats (mean difference of 62.9 mg, *p* < .001) compared to HC (Figure 1, Supplementary Data Table 1). Additionally, participants with CF had a reduced intake of iron (mean difference of −0.3 g, *p* < .001), magnesium (mean difference of −6.6 mg, *p* < .001) and an increased intake of Vitamin B12 compared to HC (mean difference of 0.3 g, *p* = .004) (Figure 1, Supplementary Data Table 1).

#### Gastrointestinal dysbiosis and inflammation in cystic fibrosis

**Figure 2.**
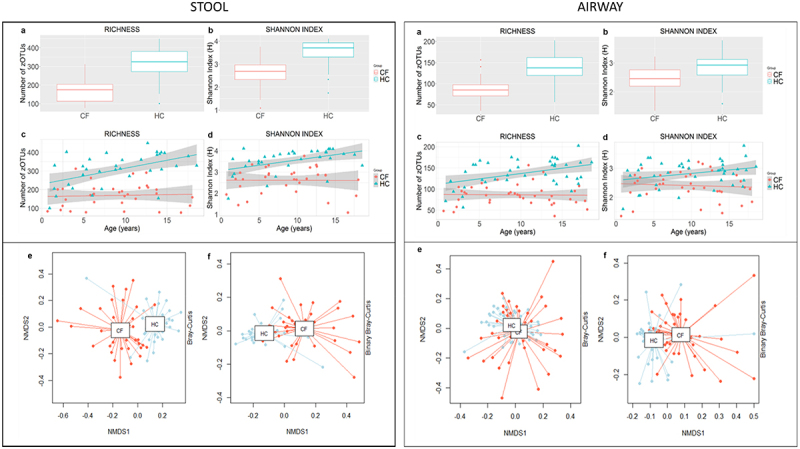
Bacterial alpha and beta diversity for stool (CF *n* = 33, HC *n* = 33) and airway samples (CF *n* = 41, HC *n* = 41). Richness was determined by number of zOTUs (a) and Shannon diversity index (b). Shaded regions represent 95% confidence intervals constructed from generalized linear models controlling for age; solid line represents mean. Beta diversity was calculated with Bray-Curtis dissimilarity to generate non-metric multidimensional scaling (NMDS) plots based on relative abundances (e) and presence/absence data (f).

Children with CF had significantly reduced bacterial richness in stool samples compared to HC (mean difference [95% CI] of −138.90 [−173.30 – −104.49], *p* < .001) (Figure 2a). The Shannon index for bacterial diversity was also significantly reduced in children with CF (mean difference [95% CI] of −0.92 [−1.19 – −0.64], (*p* < .001)) (Figure 2b). The bacterial richness and Shannon diversity continued to increase with age for HC participants but remained consistently lower throughout life for children with CF (richness estimate [SE] – 138.9 [17.9], *p < *.001) (Shannon diversity estimate [SE] – 0.92 [0.14], *p < *.001). CF and HC cohorts also had different GI microbiota based on relative abundance (*p* < .001) and presence/absence data (*p* < .001) (Figure 2e-f). ANCOM analysis (FDR of <0.05) revealed significant differences in the relative abundances of 31 genus-level bacterial taxa between CF and HC participants. CF had a relative reduction of *Akkermansia, Faecalibacterium, Alistipes, Christensenellaceae R7 Group* and species from *Ruminococcaceae* and *Lachnospiraceae*. and an increase in *Enterococcus, Enterobacter, Prevetolla, Tyzzerella 4* and *Veillonella*. (Supplementary Data Figure 3 and Table 2) Stool calprotectin levels were significantly elevated in CF participants compared to HC (median [IQR] of 120.3 [64.6 − 175.4] versus 59.4 [19.9–157.4], respectively, *p* = .044) (Supplementary Data Figure 5A).

**Figure 3.**
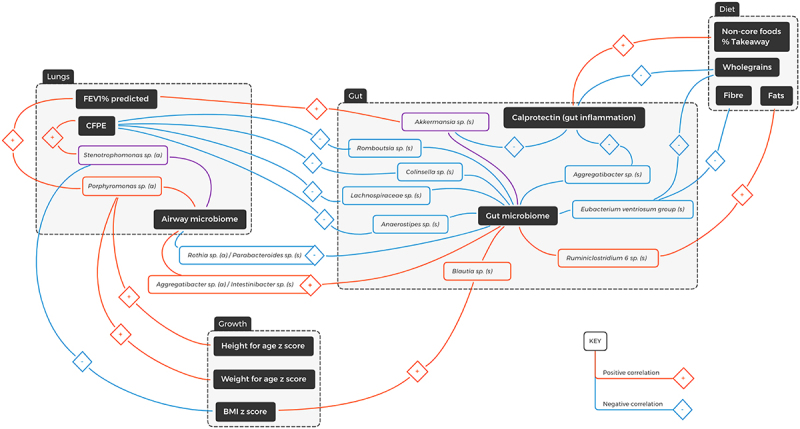
Diagrammatic representation of spearman correlations in the multisystem context of cystic fibrosis. (s) = stool genera; (a) = airway genera; FEV_1_% = percentage predicted forced expiratory volume over one second; CFPE = CF pulmonary exacerbations in the last three years. Blue line = negative correlation, red line = positive correlation, purple line = both positive and negative correlations.

**Table 2.** Clinical measurements available for CF study participants.

Within CF	All	0–10 years	>10 –18 years
***clinical analysis (n = 19)***	**19**	**8**	**11**
Age, (years, median [IQR])	11.2	7.6	13.7
Female, n (%)	10 (52.6%)	3 (37.5%)	8 (63.6%)
Pancreatic Sufficient	2	1	1
Pancreatic Insufficient	17	7	10
Genotype F508del homozygous F508del heterozygous Other	10 4 5	4 4 0	6 0 5
HAZ (SD)	0.26 (1.2)	0.28 (0.58)	0.24 (1.49)
WAZ (SD)	0.44 (0.94)	0.40 (0.52)	0.47 (1.2)
BMIZ (SD)	0.48 (0.70)	0.30 (0.60)	0.62 (0.76)
FEV_1_	2.06	1.45	2.5
FEV_1_% pred	98.9	104.1	95.0
FVC	2.50	1.70	3.08
FVC% pred	105.0	110.0	101.4
*Pseudomonas aeruginosa* infections (mean)	0.79	0.63	0.91
Detected pathogens of interest (mean)	0.74	0.13	1.18
CFPE last 3 years (mean)	1.16	0.88	1.40

#### Respiratory dysbiosis in cystic fibrosis

To examine the effect of airway sample type, we analyzed CF oropharyngeal swabs and sputum samples separately with pairwise comparisons adjusted for multiple testing. For bacterial richness, we found a significant reduction in the CF sputum (*p* < .001) and CF swab (*p* < .001) groups compared to HC, but no significant difference between the CF swab and CF sputum groups (*p* = .1). Likewise, we found a significant reduction in Shannon diversity in the CF sputum (*p* < .001) and CF swab (*p* = .003) groups compared to HC, but no significant difference between the CF swab and CF sputum groups (*p* = .097). Based on similar alpha and beta diversity results, the sputum and oropharyngeal swabs were analyzed together for further analysis. More detailed analysis of swab and sputum samples is included in the Supplementary data (Figure 2). When compared to HC, airway samples for children with CF had significantly reduced bacterial richness (mean difference [95% CI] of −51.01 [−62.61–39.41], *p* < .001) (Figure 2a), and Shannon index for diversity (mean difference [95% CI] of −0.42 [−0.61–0.24], *p* = *<* 0.001) (Figure 2b). Following a similar trend to the stool samples, airway bacterial richness and Shannon diversity continued to increase with age for HC participants but remained consistently lower throughout life for children with CF (richness estimate [SE] – 49.9 [6.2], *p <* .001) (Shannon diversity estimate [SE] – 0.41 [0.10], *p* < .001) (Figure 2c-d). Significant difference between CF and HC was also found for bacterial beta-diversity based on relative abundance (*p* < .001) and presence/absence data (*p* < .001). ANCOM analysis (FDR of <0.05) identified 26 bacterial genera reduced within the CF population including *Corynebacterium, Prevotella 2, Flavobacterium, Bergeyella, Gemella, Eucbacterium nodatum* group, *Johnsonella, Peptococcus, Peptoclostridium, Selenomonas, Selenomonas 3, Megasphera, Parvomonas, Leptotrichia, Streptobacillus, Lautropia, Alysiella, Bergeriella, Campylobacter, Aggregatibacter, Pasteurella, Treponema 2, Ruminococcaceae UCG 14*, unclassified species from *Lachnospiraceae and* candidate division *SR 1* (see also Supplementary Data Figure 4 and Table 3).

### Associations within participants with cystic fibrosis

#### Dietary intake and gastrointestinal inflammation in cystic fibrosis

For participants with CF, stool calprotectin levels positively correlated with: (i) the percentage takeaway food (r = 0.49, *q* = 0.03), and (ii) the percentage non-core food (r = 0.49, *q* = 0.03) (Supplementary data Figure 3c). Significant negative correlations for stool calprotectin were demonstrated with percentage grains (r = −0.8, *q* = <0.001), wholegrains (r = −0.57, *q* = 0.01), percentage core foods (r = −0.5, *q* = 0.03), percentage breakfast cereal (r = −0.6, *q* = 0.005), percentage proteins (r = −0.46, *q* = 0.049), total folate (r = −0.50, *q* = 0.09), thiamine (r = −0.55, *q* = 0.02), and iron (r = −0.49, *q* = 0.03) (Figure 3, Supplementary Data Figure 5C & Data Table 1).

#### Correlations between dietary intake and gastrointestinal/respiratory microbiota in cystic fibrosis

Spearman analysis of CF dietary intake and stool genera identified positive correlations between the bacterial genus *Subdoligranulum* and (i) saturated fat (r = 0.77, *q* < 0.001), (ii) trans-unsaturated fat (r = 0.70. *q* = 0.001) and (iii) retinol (r = 0.73, *q* < 0.001) (Supplementary data figure 7A). Negative correlations were identified between (i) *Subdoligranulum* and insoluble fiber (r = −0.78, *q* < 0.001), soluble fiber (r = −0.68, *q* = 0.001) and total fiber (r = −0.75, *q* = 0.003), and (ii) the *Eubacterium ventriosum group* with wholegrains (r = −0.75, *q* < 0.001), total fiber (r = −0.72, *q* < 0.001), insoluble fiber (r = −0.74, *q* < 0.001) and soluble fiber (r = −0.63, *q* < 0.001) (Figure 3, Supplementary Data Figure 7A). No significant correlations were found between dietary intake and the respiratory microbiota.

#### Gastrointestinal microbiota and clinical associations in cystic fibrosis

Negative correlations were identified between stool calprotectin concentrations and the relative abundance of the genera *Akkermansia* (r = −0.39, *q* = 0.02) and *Aggregatibacter* (r = −0.40, *q* = 0.02) (Figure 3, Supplementary Data 4B). From the age of 10 years, lung function measurements in the CF cohort were found to deteriorate while rates of CFPE increased (Table 2, Supplementary Data figure 6). Hence, for subsequent clinical association analysis, the CF cohort was divided into two groups for (0–10 years, n = 8) (>10 years, n = 11). *Akkermansia* was positively correlated with FEV_1_% predicted in the 10–18 years age group (r = 0.80, *q* = 0.049). The number of CFPE in the previous 3 years was negatively correlated with an unclassified genus within the family *Lachnospiraceae* (r = −0.81, *q* = 0.04) as well as the genera *Romboutsia* (r = −0.85, *q* = 0.02), *Anaerostipes* (r = −0.81, *q* = 0.04) and *Collinsella* (r = −0.85, *q* = 0.019) (Figure 3, Supplementary Data Figure 7C). In the 10–18 years age group, the BMI Z-score was correlated with the genera *Blautia* (r = 0.86, *q* = 0.007), and *Thalassospira* (r = 0.87, *q* = 0.001) as well as unclassified genera in the families *Christensenellaceae* (r = 0.88, *q* = 0.009), *Ruminococcaceae* (0.87, *q* = 0.001) (Figure 3, Supplementary Data 6C). No significant correlations between gastrointestinal microbiota and clinical outcome measures were found with patients under 10 years of age.

#### Respiratory microbiota and clinical associations in cystic fibrosis

Spearman analysis of CF airway genera and clinical parameters amongst the 10–18 years age group revealed a positive correlation between the bacterial genus *Stenotrophomonas* and CFPE in the last 3 years (r = 0.81, *q* = 0.004). Positive correlations were also demonstrated between the *Porphyromonas* genus and FEV_1_% (r = 0.78, *q* = 0.007), as well as weight and height z scores. (Figure 3, Supplementary Data Figure 7D). No significant correlations were found with patients under 10 years of age.

#### The microbial gut-lung axis in cystic fibrosis

Spearman analysis of CF stool (s) and airway (a) genera demonstrated significant positive correlations between *Intestinibacter* (s) and *Aggregatibacter* (a) (r = 0.54, *q* < 0.001); *Intestinibacter* (s) and *Lachnoanaerobaculum* (a) (r = 0.59, *q* < 0.001); *Prevotella 7* (s) and *Alloprevotella* (a) (r = 0.59, *q* < 0.001); and *Bacteroidales* (s) and *Corynebacterium* (a) (r = 0.60, *q* < 0.001). There was also a significant negative correlation between *Parabacteroides* (s) and *Rothia* (a) (r = −0.63, *q* < 0.001) (Figure 3, Supplementary Data Figure 7B).

## Discussion

To our knowledge, this is the first study to conduct a comprehensive dietary analysis encompassing interactions with the gastrointestinal and respiratory microbiota, and clinical outcomes in children with CF. Our results highlight several key distinctions between CF and control children. First, children with CF demonstrated a reduced relative intake of wholegrains, fiber, and resistant starch alongside increased relative intakes of total, saturated and trans fats. Second, and in line with previous studies,^11,31,40^ individuals with CF also exhibited significant respiratory and gastrointestinal dysbiosis and intestinal inflammation when compared to age and gender matched HC. Examining the intra-cohort correlations amongst the CF children, significant and multi-directional associations were identified between diet, gastrointestinal microbiota, respiratory microbiota, and clinically relevant endpoints (Figure 3). One of the key genera identified amongst this was *Akkermansia*, which was reduced in the stool of children with CF, negatively correlated with calprotectin and positively correlated with FEV_1_% pred. While *Akkermansia* is known to suppress inflammation,^41,42^ the mechanism by which this gastrointestinal organism might influence lung function remains largely unknown,^43^ but supports the concept of multi-system crosstalk and a gut-lung axis in CF. Moreover, our findings support the adage of “you are what you eat” with stool calprotectin levels positively correlated with consumption of takeaway and non-core foods, and negatively correlated with intake of grains, wholegrains and core foods.

The higher energy intake for children with CF is consistent with the recommended high energy, high fat CF diet.^4,44^ The proportional consumption of fats, predominated by trans and saturated fats corroborates the findings from Sutherland *et al.*,^5^ indicating children with CF are likely to meet energy recommendations through energy-dense, nutrient-poor diets. While patients with CF have historically been at risk of malnutrition, more recent studies have shown an increasing prevalence of overnutrition and obesity.^45^ Consistent with this, many of the stool bacterial genera associated with the CF cohort in our study have established links to obesity and adult-onset complications of an energy-dense, nutrient-poor diet.^5^ The *Christensenellaceae* family and its genus *Christensenellaceae R7* group, which were reduced in the CF cohort, have established negative correlations with obesity and indices of cardiometabolic risk including central adiposity and visceral fat mass.^46^ The *Eubacterium coprostanoligenes* group, which was also reduced in CF, have been implied in animal models to reduce plasma cholesterol levels via the conversion of cholesterol to coprostanol, which is then faecally excreted.^47,48^ Fiber and wholegrain intake were both negatively correlated with the relative abundance of the *Eubacterium ventriosum* group, which has a known positive association with obesity.^49,50^ As the mean age of the global CF population is relatively young, there is little longitudinal evidence on the implications of obesity and a nutrient poor diet within this population later in life. However, the high fat CF diet warrants a contemporary review in the context of an aging CF population, the greater risk of colorectal cancer (CRC) in young adults with CF^51^ and the issues of weight gain associated with cystic fibrosis transmembrane conductance regulator modulator therapies.^52^

Gastrointestinal inflammation has become a recognized pathological hallmark of CF.^14–16^ The higher stool calprotectin concentrations identified for the CF cohort were therefore not unexpected. Within CF, stool calprotectin levels were negatively correlated with the intake of wholegrains and core foods. The proportional intake of wholegrains was reduced in the CF cohort, along with starch, resistant starch, and fiber, all known to have anti-inflammatory implications for gastrointestinal health. In contrast, a positive correlation was found between stool calprotectin and the intake of takeaway and non-core (i.e. junk) foods. The link between diet and inflammation is consistent with findings in the broader community^53–56^ and within other inflammatory conditions including ulcerative colitis^57^ and CRC.^58^ Fritsch et al. demonstrated that low-fat, high-fiber diets can reduce markers of inflammation and improve quality of life.^57^ In a similar vein, a review of 45 meta-analyses indicated that an increased intake of dietary fiber, along with calcium and yogurt, and a reduced intake of alcohol and red meat was associated with lower CRC rates.^58^ Our findings demonstrate the relative deficiency of fiber-based foods, high consumption of saturated fats and elevated stool calprotectin levels amongst the CF cohort, and support the hypothesis of the CF diet as a potential contributor to gastrointestinal inflammation and future CRC risk.

Specific gastrointestinal bacterial genera were increased (*Enterococcus, Enterobacter, Lachnoclostridium*) or decreased (*Akkermansia, Faecalibacterium* and *Alistipes*) in CF. Based on evidence within the existing literature, these alterations may promote inflammation, pathogenic colonization and ultimately malignancy of the intestinal tract. *Enterococcus* species are primarily recognized as gastrointestinal commensals, yet the genus also harbors many prevalent multidrug-resistant nosocomial pathogens, known to densely colonize the gut following antibiotic treatment.^59,60^ *Enterobacter* is associated with a variety of chronic inflammatory conditions, including inflammatory bowel disease, infection, CRC and food allergies.^61^ *Lachnoclostridium* has recently been identified as a novel stool biomarker for colorectal adenoma and cancer.^62^ *Akkermansia* is known to have protective anti-obesity and anti-inflammatory implications,^41,63,64^ while *Faecalibacterium* has a role in attenuating intestinal inflammation and mediating host immune response through butyrate production.^65^ The *Alistipes* genus is known to be involved in the production of succinic acid, which improves glucose homeostasis via intestinal gluconeogenesis in murine studies.^66^ However, it also is important to note that there is contrasting evidence indicating that *Alistipes* may be protective against liver fibrosis, colitis, cancer immunotherapy and cardiovascular disease, but may alternatively play a pathogenic role in CRC.^67^

The specific bacterial genera that are reduced in the CF airway microbiota likewise have inflammatory and colonization implications for the lungs. *Megasphaera* is a short chain fatty acid (SCFA) – producing respiratory commensal with a potential role in reducing airway inflammation and pathogen colonization in the setting of chronic obstructive pulmonary disease (COPD).^68^ *Corynebacterium* has been demonstrated to shift *Staphylococcus aureus* from a virulent to commensal state in the airway milieu.^69^ And finally, *Selenomonas* and *Selenomonas 3*, both of which are decreased in the CF group, are considered to be markers of healthy respiratory flora.^70^ No direct correlations were found between the respiratory microbiota and the CF diet or stool calprotectin. These results suggest that respiratory microbiome alterations are more likely to be driven by CF disease-specific factors, or in-directly modulated via the GI microbiota through mechanisms such as SCFA and systemic inflammation.

Our results identified several axial relationships between the altered CF gastrointestinal microbiota, respiratory microbiota and clinical health outcomes which support a potential role of the gut-lung axis in disease modification and pulmonary outcomes (Figure 3). For example, gastrointestinal microbiota positively correlated with lung function (FEV_1_% predicted). The number of recent CFPE was negatively correlated to the butyrate-producing gastrointestinal bacteria *Lachnospiraceae,^71^Romboutsia^72^ and Anaerostipes,^73^* and positively correlated to the airway genus *Stenotrophomonas maltophilia*, a known CF pulmonary pathogen that can increase morbidity and mortality with chronic infection.^74,75^ Further correlations between the gastrointestinal and respiratory microbiota included the opportunistic respiratory pathogen *Rothia*, prominent in immunocompromised populations,^76^ negatively correlated with stool-bacterium *Parabacteroides*, a gut commensal with anti-inflammatory implications,^77,78^ and *Porphyromonas*, a commensal and part of the salivary microbiota in healthy individuals^79^ positively correlated with FEV_1_% predicted as well as weight and height z scores.

This study provides a cross-sectional snapshot of the constituents of and correlations between the gastrointestinal and respiratory milieu in CF, longitudinal analysis will ultimately be required to evaluate the significance of these findings in a temporal context. Likewise, the significant correlations identified, do not prove causality and future mechanistic models are needed to confirm diet-gut-lung effects. This study was also limited by the single-center recruitment, relatively small sample size and inherent limitations to data collection in a pediatric population. Sputum samples were prioritized but dependent on ability to expectorate and what each individual child could tolerate. Based on similar alpha and beta diversity results, the resulting sputum and oropharyngeal swabs were analyzed together. Limited sample numbers prevented meaningful sub-sample correlation analysis between CF swab and CF sputum microbiota samples. Further, only children >5 years old were able to perform lung function testing, limiting the available clinical data for analysis. The dietary analyses completion rates were limited, presumably through existing treatment and survey burden. Antibiotic usage is also a known confounder for microbial analysis,^80^ however, as antibiotic therapy is an integral and essential part of CF management it was impractical to exclude antibiotic users from participating in the current prospective study.

Despite the limitations, clear alterations were demonstrated in the respiratory and gastrointestinal milieu in CF, evidenced by marked microbial dysbiosis and intestinal inflammation when compared to a control cohort. This was seen in conjunction with significant differences in relative dietary intake between study groups. The CF cohort provided additional evidence to support the concept of a gut-lung axis between the altered respiratory and gastrointestinal microbiota, and correlations with lung function and pulmonary exacerbations. Diet has direct links to SCFA synthesis,^81–83^ inflammation^26–28,53,57^ and is an established modulator of gastrointestinal microbiota.^84^ Our results, combined with the established modulatory role of diet, position the altered CF diet as a promising modifiable component of the proposed gut-lung-axis. The drivers of inflammation and dysbiosis in CF are complex and multifactorial.^85^ Dietary interventions are unlikely to completely restore the GI or respiratory milieu. Nonetheless, increasing intakes of wholegrains, fiber and resistant starch may reduce inflammation and improve microbiota composition and consequently warrant further investigation within the framework of the recommended CF diet.

## Supplementary Material

Supplemental Material

## Data Availability

Data presented in this research paper was obtained according to the Evaluating the Alimentary and Respiratory Tracts in Health and Disease (EARTH) Research Program, registered in clinicaltrials.gov (NCT04071314). The datasets generated and analysed for the current study are available in the figshare repository, DOI: 10.6084/m9.figshare.19416830.
