# Supplementary material for: Diet and the gut-lung axis in cystic fibrosis – direct & indirect links
Source: Gut Microbes. 2022 Dec 27;15(1):2156254. doi: 10.1080/19490976.2022.2156254 (PMC9809969; doi:10.1080/19490976.2022.2156254)
Supplement: Supplemental Material [file KGMI_A_2156254_SM0703.docx]

**Supplementary Data**

**Table 1: Summary statistics of average daily intake of macro and micronutrients obtained from Australian Easting Survey for CF and HC cohorts.**

|  | **CF** | | | **HC** | | | | |  |
| --- | --- | --- | --- | --- | --- | --- | --- | --- | --- |
|  | **median** | quartile 1 | quartile 3 | **median** | quartile 1 | | | quartile 3 | *P -* value |
| **Macronutrients** |  |  |  |  | |  |  | |  |
| Total Kilojoules | **11531.0** | 6856.5 | 14376.5 | **7331.0** | | 5788.8 | 9067.0 | | 0.008** |
| Moisture (g) | **2880.8** | 1534.1 | 3460.9 | **2134.5** | | 1903.0 | 2352.6 | | 0.134 |
| Protein (g) | **118.4** | 59.9 | 144.3 | **69.2** | | 52.5 | 95.5 | | 0.011* |
| Fat (g) | **127.3** | 71.6 | 145.9 | **64.8** | | 52.5 | 77.4 | | 0.001** |
| Carbohydrate (g) | **277.1** | 185.0 | 383.3 | **217.9** | | 148.7 | 256.3 | | 0.029* |
| Starch (g) | **132.1** | 86.1 | 179.4 | **117.5** | | 88.0 | 142.6 | | 0.461 |
| Sugar (g) | **161.9** | 98.4 | 221.5 | **93.7** | | 74.1 | 122.2 | | 0.006* |
| Fibre (g) | **23.8** | 17.9 | 34.3 | **23.7** | | 21.5 | 28.4 | | 0.822 |
| **g/mg/ug per 1000 kJ** | | |  |  | |  |  | |  |
| Protein (g) | **9.5** | 9.1 | 9.9 | **9.7** | | 8.6 | 10.2 | | 0.980 |
| Fat (g) | **10.3** | 9.5 | 10.8 | **8.4** | | 7.9 | 9.8 | | 0.003** |
| Carbohydrate (g) | **25.7** | 24.5 | 27.3 | **28.3** | | 25.7 | 31.2 | | 0.030* |
| Starch (g) | **11.1** | 10.2 | 13.1 | **15.7** | | 13.0 | 17.0 | | 0.007* |
| Sugar (g) | **14.4** | 13.2 | 15.0 | **12.9** | | 11.7 | 14.6 | | 0.189 |
| Added sugars (g) | **4.8** | 3.9 | 5.9 | **3.4** | | 2.6 | 5.7 | | 0.233 |
| Cholesterol (mg) | **29.1** | 25.6 | 35.0 | **28.2** | | 24.7 | 40.6 | | 0.964 |
| Trytophan (mg) | **105.7** | 101.6 | 112.9 | **110.4** | | 100.9 | 112.6 | | 0.642 |
| Sat fat (g) | **4.6** | 4.0 | 4.9 | **3.3** | | 2.9 | 3.9 | | <0.001*** |
| Mono fat (g) | **3.7** | 3.4 | 4.1 | **3.2** | | 2.9 | 3.9 | | 0.053 |
| Poly fat (g) | **1.1** | 0.9 | 1.2 | **1.1** | | 1.0 | 1.2 | | 0.425 |
| Linoleic (mg) | **0.9** | 0.8 | 1.0 | **1.0** | | 0.9 | 1.0 | | 0.343 |
| epa (mg) | **6.7** | 4.1 | 8.9 | **4.6** | | 4.1 | 7.9 | | 0.538 |
| dpa (mg) | **7.8** | 7.3 | 9.5 | **6.4** | | 4.6 | 7.6 | | 0.404 |
| dha (mg) | **7.4** | 3.3 | 12.4 | **8.5** | | 4.3 | 12.4 | | 0.798 |
| Omega3 (mg) | **23.0** | 15.1 | 29.8 | **19.2** | | 13.5 | 27.5 | | 0.461 |
| Trans fat (mg) | **212.1** | 177.8 | 229.4 | **149.2** | | 123.0 | 194.2 | | <0.001*** |
| Wholegrains (g) | **2.8** | 1.7 | 3.3 | **4.5** | | 3.6 | 7.2 | | 0.006* |
| Resistant starch (g) | **0.2** | 0.2 | 0.4 | **0.5** | | 0.3 | 0.7 | | 0.007* |
| Total fibre (g) | **0.9** | 0.8 | 1.2 | **1.4** | | 1.1 | 1.6 | | 0.001** |
| Insol fibre (g) | **0.6** | 0.5 | 0.8 | **0.9** | | 0.7 | 1.1 | | 0.001** |
| Soluble fibre (g) | **0.3** | 0.3 | 0.4 | **0.4** | | 0.4 | 0.5 | | 0.006* |
| Ash (μg) | **1.8** | 1.7 | 1.9 | **1.8** | | 1.7 | 1.9 | | 0.99 |
| Beta Carotene (μg) | **233.8** | 177.0 | 325.7 | **319.1** | | 258.5 | 470.4 | | 0.07 |
| Vit A (μg) | **283.7** | 219.6 | 424.0 | **402.5** | | 342.0 | 591.8 | | 0.066 |
| Retinol eq (μg) | **97.0** | 78.2 | 115.7 | **107.5** | | 88.6 | 124.5 | | 0.400 |
| Thiamin (mg) | **0.2** | 0.1 | 0.2 | **0.2** | | 0.1 | 0.3 | | 0.119 |
| Riboflavin (mg) | **0.3** | 0.2 | 0.3 | **0.2** | | 0.2 | 0.4 | | 0.578 |
| Niacin_Eq (μg) | **3.9** | 3.5 | 4.3 | **3.9** | | 3.7 | 4.5 | | 0.233 |
| Dietary Folate (μg) | **54.7** | 48.9 | 61.4 | **69.9** | | 59.5 | 81.9 | | 0.051 |
| Vit_B6 (μg) | **0.2** | 0.1 | 0.2 | **0.2** | | 0.1 | 0.2 | | 0.868 |
| Vit_B12 (μg) | **0.6** | 0.5 | 0.7 | **0.4** | | 0.3 | 0.5 | | 0.004** |
| Vit_C (μg) | **15.3** | 9.4 | 19.3 | **16.6** | | 12.4 | 18.5 | | 0.538 |
| Vit_E (μg) | **1.0** | 0.8 | 1.2 | **1.0** | | 1.0 | 1.2 | | 0.159 |
| Calcium (mg) | **124.9** | 114.6 | 138.1 | **94.7** | | 82.6 | 134.7 | | 0.111 |
| Iodine (mg) | **20.5** | 16.5 | 23.9 | **16.7** | | 14.3 | 21.6 | | 0.257 |
| Iron (mg) | **1.0** | 0.8 | 1.1 | **1.3** | | 1.1 | 1.5 | | <0.001*** |
| Magnesium (mg) | **35.7** | 33.3 | 39.5 | **42.3** | | 39.7 | 47.3 | | <0.001*** |
| Phosphorus (mg) | **172.1** | 163.0 | 184.6 | **167.6** | | 152.2 | 173.0 | | 0.298 |
| Potassium (mg) | **359.0** | 320.2 | 379.9 | **359.6** | | 339.4 | 389.7 | | 0.479 |
| Selenium (mg) | **8.0** | 6.6 | 9.9 | **9.1** | | 8.4 | 10.9 | | 0.057 |
| Sodium (mg) | **218.3** | 196.4 | 227.8 | **219.2** | | 182.4 | 267.5 | | 0.869 |
| Zinc (mg) | **1.3** | 1.1 | 1.4 | **1.3** | | 1.2 | 1.4 | | 0.663 |
| **Energy from food groups** | | |  |  | |  |  | |  |
| % Core | **64.0** | 60.5 | 69.0 | **76.0** | | 65.0 | 79.5 | | 0.025* |
| % non-core | **36.0** | 31.0 | 39.5 | **24.0** | | 20.5 | 37.3 | | 0.025* |
| % Protein | **16** | 16 | 17 | **16.5** | | 15.0 | 17.5 | | 0.877 |
| % Carbohydrate | **44** | 42.5 | 47 | **48.5** | | 44.5 | 53.5 | | 0.031* |
| % Fats | **40.0** | 36.5 | 42.0 | **32.5** | | 31.0 | 39.0 | | 0.003** |
| % Saturated fats | **18.0** | 15.5 | 19.0 | **13** | | 11.5 | 16.5 | | <0.001*** |
| % Poly fats | **4.0** | 4.0 | 5.0 | **4** | | 4 | 5 | | 0.506 |
| % Mono fats | **14.0** | 13.0 | 16.0 | **12.5** | | 11.5 | 15.5 | | 0.071 |
| % Added sugars | **8.0** | 6.5 | 10.0 | **6** | | 4 | 10 | | 0.194 |
| % Veg | **6** | 4.5 | 8 | **5.5** | | 3 | 9 | | 0.99 |
| % Fruit | **6** | 4 | 9 | **10** | | 8 | 14 | | 0.008* |
| % Meat | **11** | 8 | 13 | **7** | | 6 | 13 | | 0.206 |
| % Grains | **14** | 9.5 | 17 | **23.5** | | 18 | 28 | | <0.001*** |
| % Dairy | **23** | 20.5 | 30.5 | **12.5** | | 7 | 24 | | 0.013* |
| % Sweet drink | **2** | 0 | 4.5 | **0** | | 0 | 1 | | 0.006* |
| % Packaged snack | **6** | 4.5 | 7 | **3** | | 1 | 4 | | 0.004** |
| % Confectionary | **6** | 3 | 8 | **3.5** | | 2 | 9.5 | | 0.463 |
| % Baked products | **4** | 1.5 | 8.5 | **3.5** | | 1.5 | 4.5 | | 0.367 |
| % Takeaway | **9** | 6.5 | 10.5 | **8** | | 6.5 | 11.5 | | 0.915 |
| % Condiments | **2** | 1 | 2.5 | **2** | | 1 | 3 | | 0.573 |
| % Fatty meats | **2** | 1 | 3 | **1** | | 0 | 3 | | 0.179 |
| % Breakfast cereal | **3** | 0.5 | 6.5 | **8** | | 3 | 12 | | 0.026* |
| % Meals with veg | **7** | 3.5 | 9.5 | **5** | | 2.5 | 8 | | 0.206 |
| % Meals no veg | **1** | 0.5 | 2.5 | **1** | | 0 | 2.25 | | 0.707 |

Primary macronutrients are listed in total grams/day. Macro and micronutrients listed as g/mg/μg per 1000kJ/day. The total energy obtained from individual food groups is listed as a percentage (%) of total kJ/day. The food groupings used in the ACAES dietary survey^35^ include; Core foods: breads and cereals, fruit, vegetables and salad, dairy and alternatives, meat and alternatives, as well as tea and coffee; non-core foods: sweetened drinks, packaged snacks, confectionary, baked/sweet products, fried/takeaway food, spreads and sauces, fatty meats, alcohol and clear or creamy soups; Takeaway: crumbed chicken, crumbed fish, tacos, hamburgers, pizza, pies, hot dogs, savoury pastries, hash browns, takeaway fries and home fries


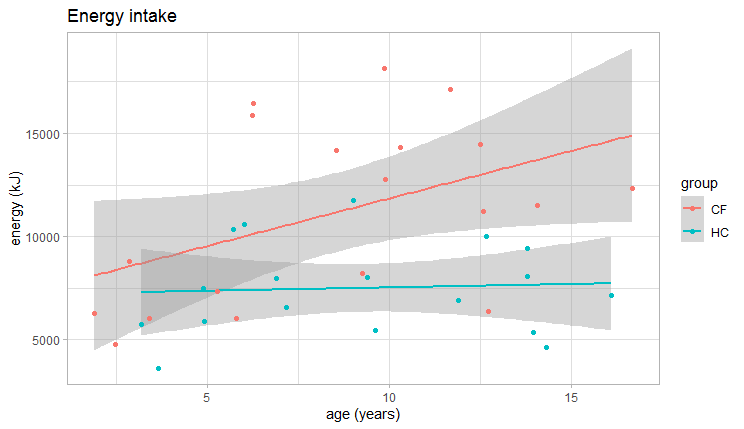


Figure 1: Energy intake (kJ) between CF (red) and HC (blue) groups. Shaded regions represent 95% confidence intervals constructed from generalised linear models controlling for age; solid line represents mean.

***CF swab vs CF sputum airway samples***

To examine the effect of airway sample type, we analysed CF swab and sputum samples separately with pairwise comparisons adjusted for multiple testing. The bacterial richness compared to HC (mean [SD] = 137.26 [31.67]) was significantly reduced in the CF sputum (mean [SD] = 73.56 [21.22], *p* < 0.001) and CF swab (mean [SD] = 90.46 [24.84], *p* < 0.001) groups, but there was no significant difference between the CF swab and sputum groups (*p* = 0.1). Likewise, we found a significant reduction in Shannon diversity in the CF sputum (mean [SD] = 2.19 [0.53], *p* < 0.001) and CF swab (mean [SD] 2.48 [0.45], *p* = 0.003) groups compared to HC, but no significant difference between the CF swab and sputum groups (*p* = 0.097). Consistent with the combined airway analysis, the beta diversity was significantly different between HC and CF swab and Sputum samples based on binary and relative abundance data (*p* < 0.001, *p* < 0.001), but not significant between swab and sputum sample groups. However, unlike the combined airway analysis, we did find a significant difference in age between CF swab and CF sputum samples with binary and relative abundance data (*p* = 0.02, *p* = 0.003), reflecting the increased tolerance for sputum sample collection with increasing age.


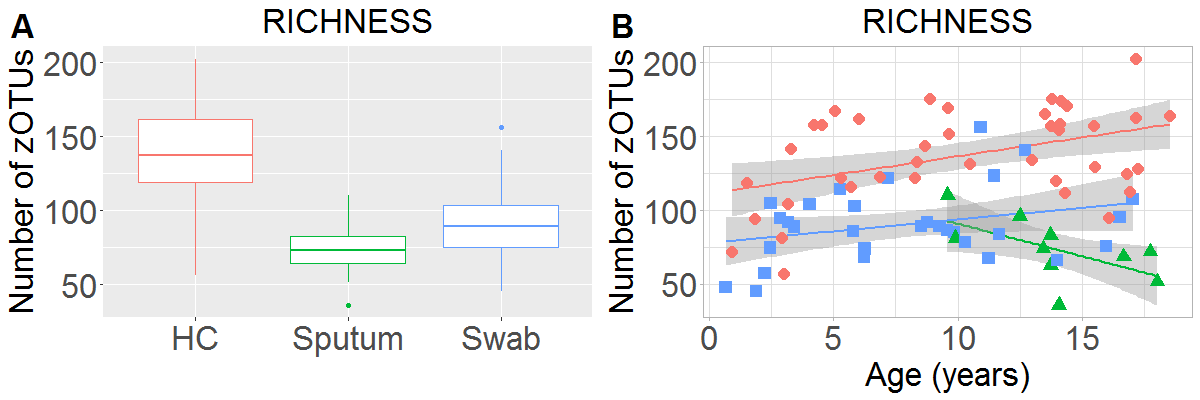
Figure 2. Bacterial richness in airway samples for HC and CF swab and CF sputum. Shaded regions represent 95% confidence intervals constructed from generalised linear models controlling for age; solid line represents mean.


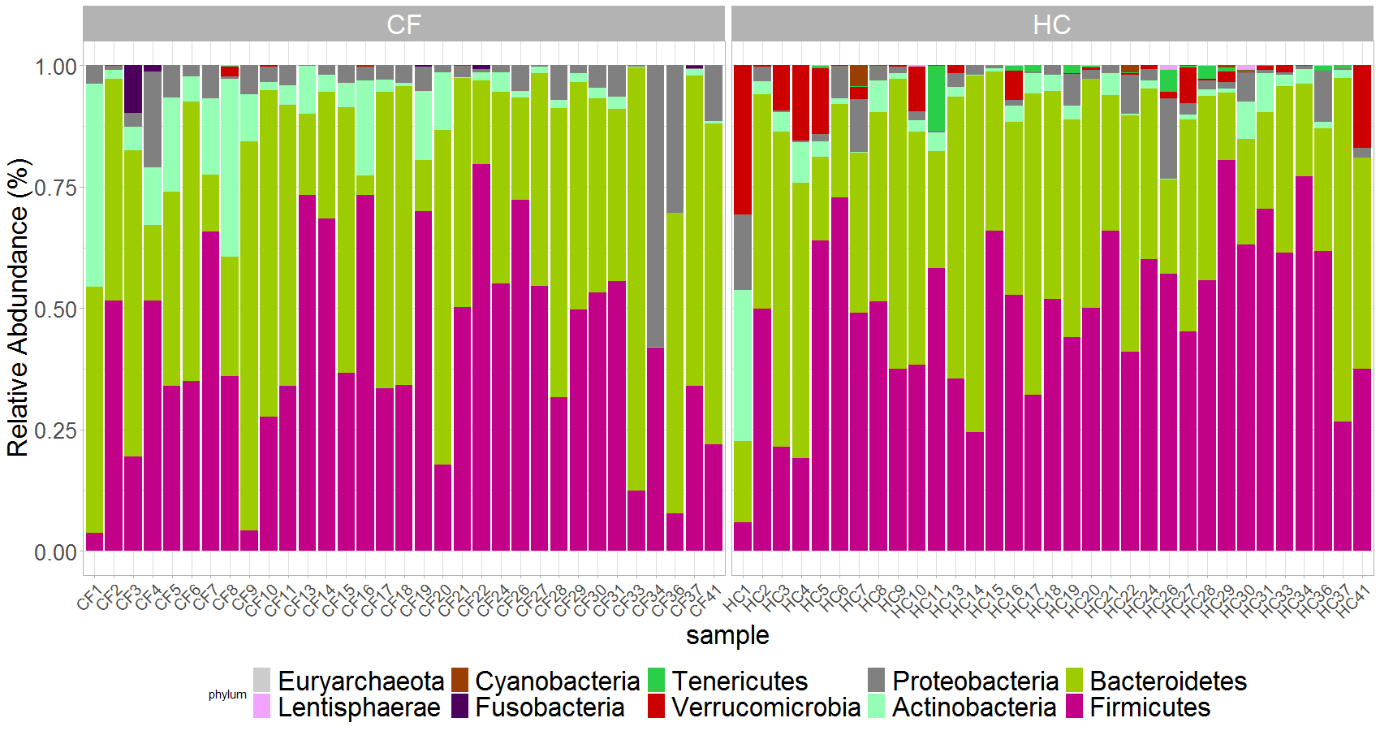


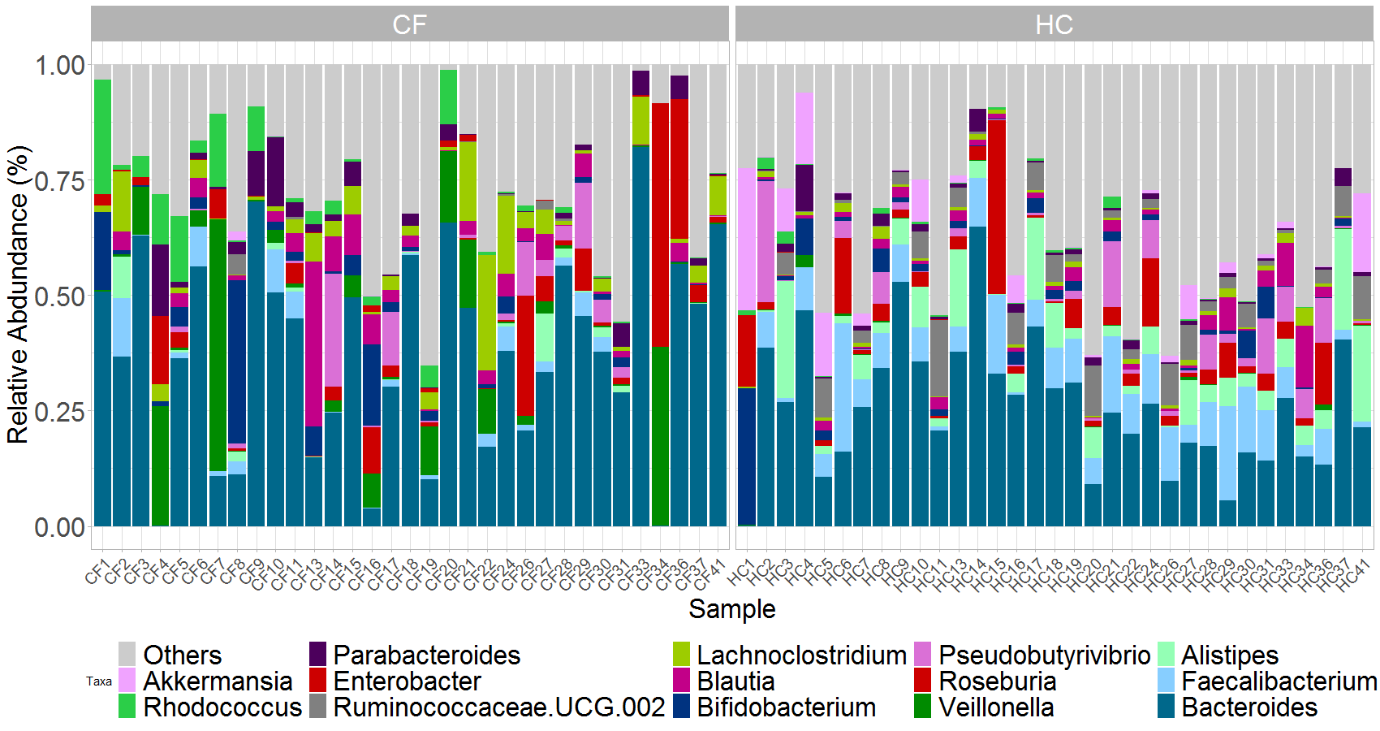


Figure 3: The relative abundance of stool bacterial phyla and the top 14 genera in CF and HC subjects. Subjects are ordered in increasing age (left to right).


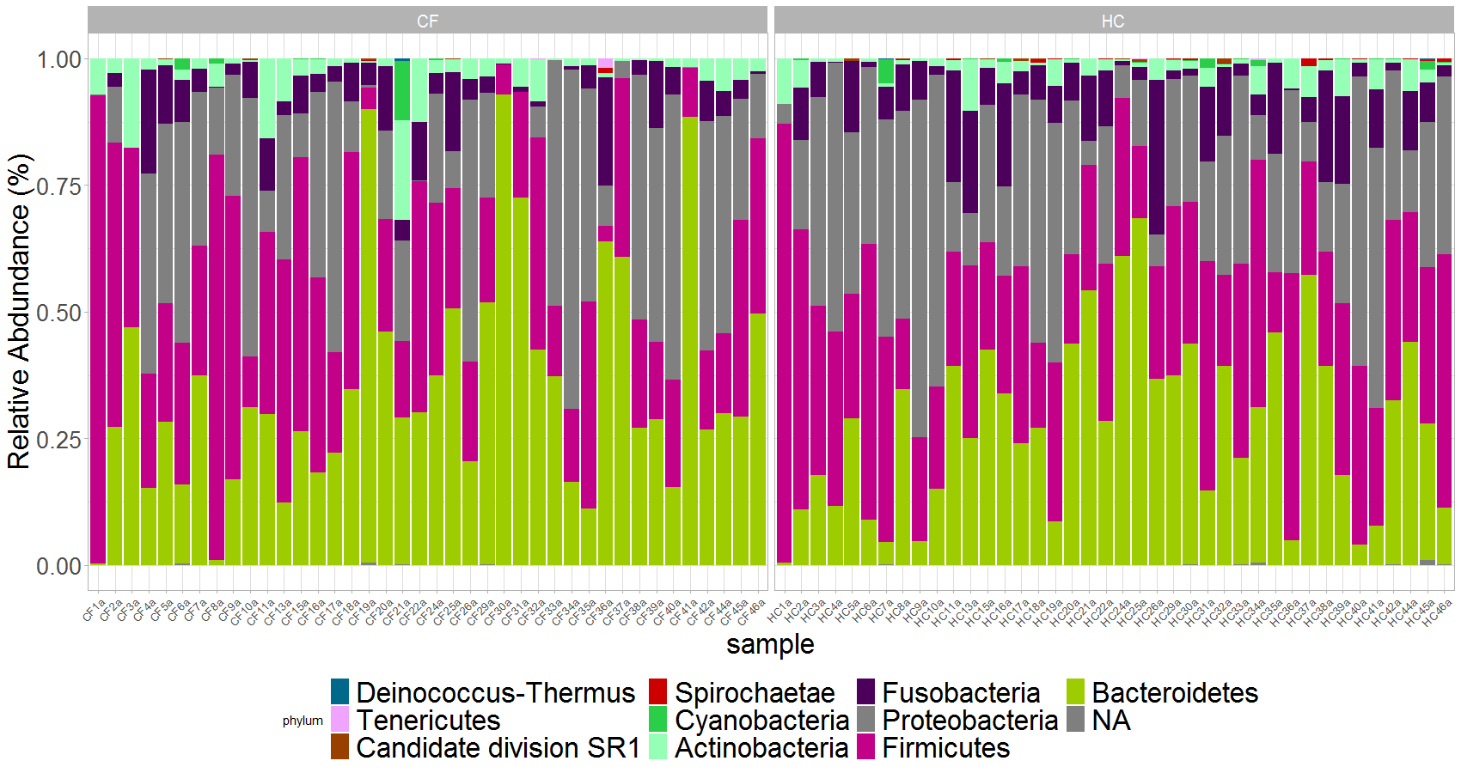


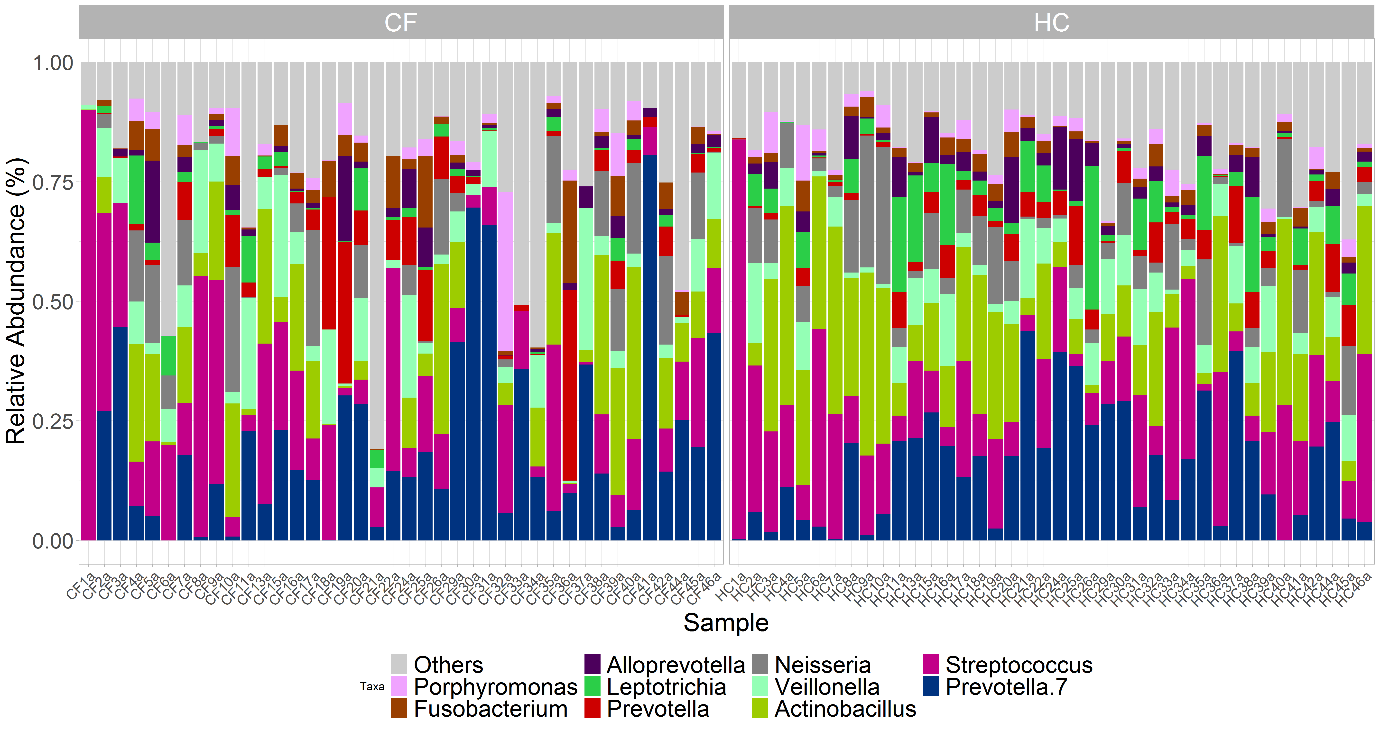


Figure 4: The relative abundance of airway bacterial phyla and the top 10 genera in CF and HC subjects. Subjects are ordered in increasing age (left to right).

| **PHYLUM** | **CLASS** | **ORDER** | **FAMILY** | **GENUS** |
| --- | --- | --- | --- | --- |
| Actinobacteria | Actinobacteria | ↑ Corynebacteriales | ↑ Nocardiaceae | *↑ Rhodococcus* |
| Bacteroidetes | Bacteroidia | Bacteroidales | Odoribacteraceae | *↓ Odoribacter* |
|  |  |  | Prevotellaceae | *↑ Prevotella 7* |
|  |  |  | ↓ Rikenellaceae | *↓ Alistipes* |
| Firmicutes | Clostridia | Clostridiales | ↓ Christensenellaceae | *↓ Christensenellaceae R 7 group* |
|  |  |  | ↓ Clostridiales vadin BB60 group |  |
|  |  |  | Eubacteriaceae | *↓ Eubacterium coprostanoligenes group* |
|  |  |  | ↓ Family XIII | *↓ Family XIII AD3011 group* |
|  |  |  | Lachnospiraceae | *↓ Coprococcus 2* |
|  |  |  |  | *↑ Lachnoclostridium* |
|  |  |  |  | *↓ Lachnospiraceae FCS020 group* |
|  |  |  |  | *↓ Lachnospiraceae NC2004 group* |
|  |  |  |  | *↓ Lachnospiraceae NK4A136 group* |
|  |  |  |  | *↑ Tyzzerella 4* |
|  |  |  | Hungateiclostridiaceae | *↓ Ruminiclostridium 6* |
|  |  |  | ↓ Ruminococcaceae | *↓ Anaerotruncus* |
|  |  |  |  | *↓ Faecalibacterium* |
|  |  |  |  | *↓ Ruminococcaceae NK4A214 group* |
|  |  |  |  | *↓ Ruminococcaceae UCG 002* |
|  |  |  |  | *↓ Ruminococcaceae UCG 003* |
|  |  |  |  | *↓ Ruminococcaceae UCG 005* |
|  |  |  |  | *↓ Ruminococcaceae UCG 010* |
|  |  |  |  | *↓ Ruminococcaceae UCG 013* |
|  |  |  |  | *↓ Ruminococcaceae UCG 014* |
|  |  |  |  | *↓ Ruminococcus 1* |
|  |  |  |  | *↓ Subdoligranulum* |
|  | ↑ Bacilli | ↑ Lactobacillales | ↑ Enterococcaceae | *↑ Enterococcus* |
|  | Erysipelotrichia | Erysipelotrichales | Erysipelotrichaceae | *↓ Erysipelotrichaceae UCG 003* |
|  | Negativicutes | ↑ Selenomonadales |  |  |
|  |  | Veillonellales | Veillonellaceae | *↑ Megasphaera* |
|  |  |  |  | *↑ Veillonella* |
| ↓ Lentisphaerae |  |  |  |  |
| Proteobacteria | Gammaproteobacteria | ↑ Enterobacterales | ↑ Enterobacteriaceae | *↑ Enterobacter* |
| ↓ Tenericutes | ↓ Mollicutes | ↓ Mollicutes RF9 | ↓ Mollicutes RF9 unc |  |
| ↓ Verrucomicrobia | ↓ Verrucomicrobiae | ↓ Verrucomicrobiales | Akkermansiaceae | *↓ Akkermansia* |
|  |  |  | ↓ Verrucomicrobiaceae |  |

Table 2: Significantly different bacterial taxa between CF and HC stool samples

| **PHYLUM** | **CLASS** | **ORDER** | **FAMILY** | **GENUS** |
| --- | --- | --- | --- | --- |
| Actinobacteria | Actinobacteria | Corynebacteriales | Corynebacteriaceae | *↓ Corynebacterium* |
| Bacteroidetes | Bacteroidia | Bacteroidales | Prevotellaceae | *↓ Prevotella 2* |
|  | Flavobacteriia | Flavobacteriales | Flavobacteriaceae | *↓ Flavobacterium* |
|  |  |  | Weeksellaceae | *↓ Bergeyella* |
| *↓* Candidate division SR1 | *↓* Candidate division SR1 | *↓* Candidate division SR1 | *↓* Candidate division SR1 | *↓ SR 1 UNC* |
| Firmicutes | Bacilli | ↓Bacillales | ↓Bacillales | *↓ Gemella* |
|  | Clostridia | Clostridiales | Eubacteriaceae | *↓ Eubacterium nodatum group* |
|  |  |  | Lachnospiraceae | *↓ Johnsonella* |
|  |  |  |  | *↓ Lachnospiraceae UCG 008* |
|  |  |  |  | *↓ Lachnospiraceae UNC* |
|  |  |  | ↓Peptococcaceae | *↓ Peptococcus* |
|  |  |  | Peptostreptococcaceae | *↓ Peptoclostridium* |
|  |  |  | ↓Ruminococcaceae | *↓ Ruminococcaceae UCG 14* |
|  | Negativicutes | Selenomonadales | Selenomonadaceae | *↓ Selenomonas* |
|  |  | Selenomonadales | Selenomonadaceae | *↓ Selenomonas 3* |
|  |  | Veillonellales | Veillonellaceae | *↓ Megasphaera* |
|  | Tissierellia | Tissierellales | Peptoniphilaceae | *↓ Parvimonas* |
| Fusobacteria | Fusobacteriia | Fusobacteriales | Leptotrichiaceae | *↓ Leptotrichia* |
|  |  |  |  | *↓ Streptobacillus* |
| Proteobacteria | Betaproteobacteria | ↓Burkholderiales | Burkholderiaceae | *↓ Lautropia* |
|  |  | Neisseriales | Neisseriaceae | *↓ Alysiella* |
|  |  |  |  | *↓ Bergeriella* |
|  | Epsilonproteobacteria | Campylobacterales | ↓Campylobacteraceae | *↓ Campylobacter* |
|  | Gammaproteobacteria | Pasteurellales | Pasteurellaceae | *↓ Aggregatibacter* |
|  |  |  |  | *↓ Pasteurella* |
| *↓* Spirochaetes | ↓Spirochaetes | ↓Spirochaetales | Spirochaetaceae | *↓ Treponema 2* |

Table 3: Significantly different bacterial taxa between CF and HC airway samples

Arrows indicate if the relative abundance of each taxa is higher (↑) or lower (↓) in CF compared with HC populations. Taxa in grey describe phylogeny and were not significantly different.


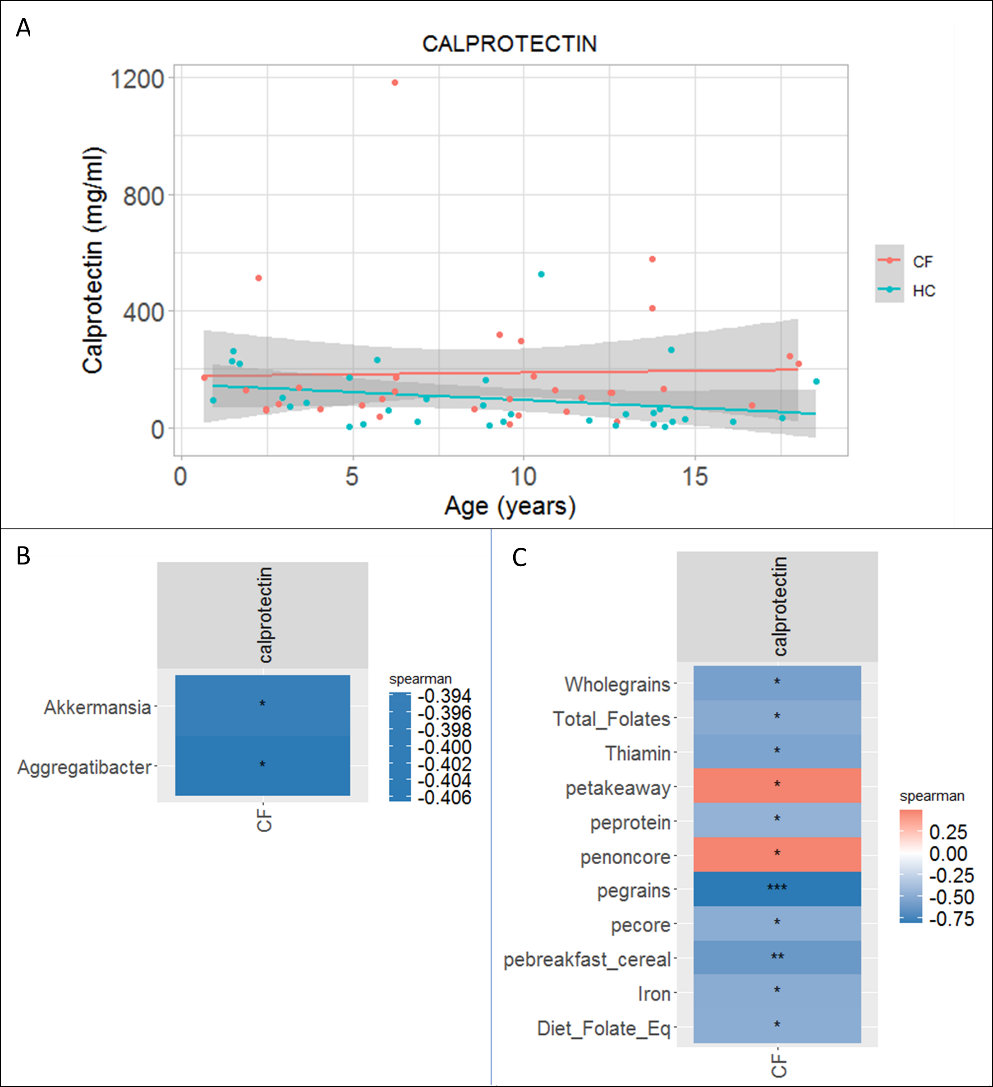


Figure 5: Fecal calprotectin measurements and correlations with gastrointestinal microbiota and dietary intake. A) Scatterplot of fecal calprotectin levels between CF (red) and HC (blue) groups. Shaded regions represent 95% confidence intervals constructed from generalized linear models controlling for age; solid line represents mean. B) Significant corrleations identified between calprotectin and stool taxa identified to the genera level for the CF cohort. C) Significant corrleations identified between calprotectin and dietary intakes for CF cohort. *P<0.05, **P<0.01, ***P<0.001


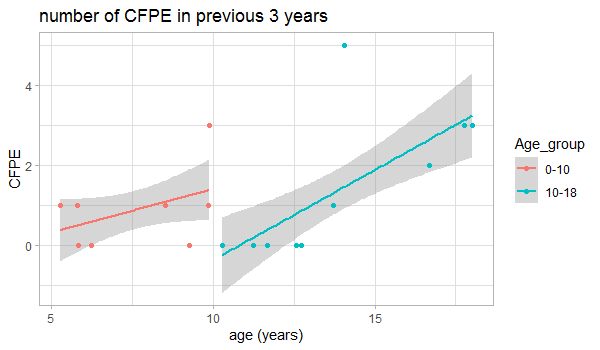


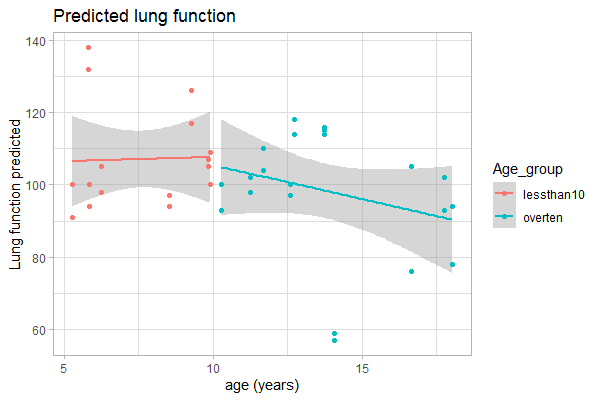


Figure 6: Physician confirmed CFPE and pulmonary function (spirometry) across age. (6A) The rate of CFPE in the last three years increases after 10 years of age. (6B) Predicted lung function is stable until 10 years of age, then declines.


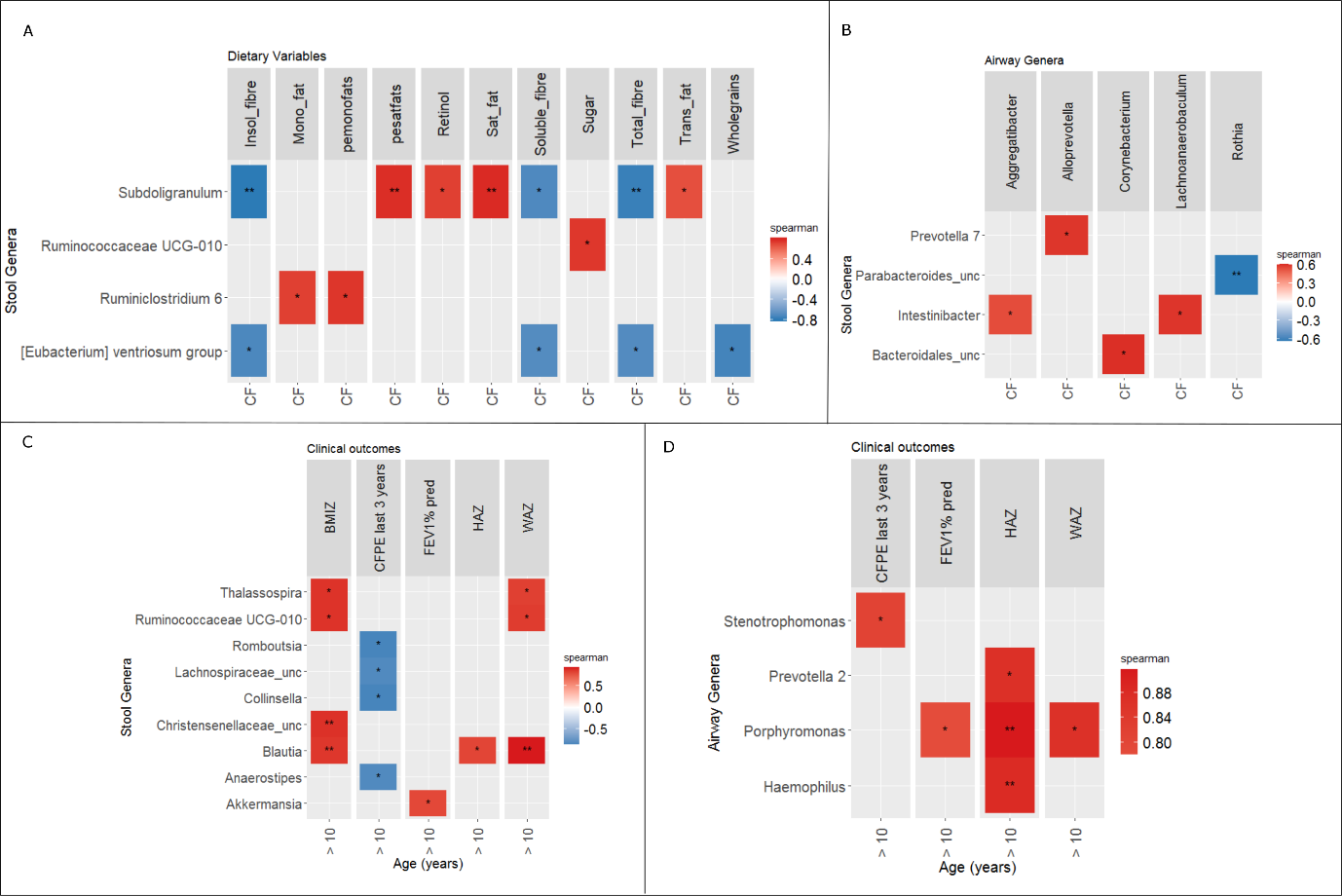


Figure 7: Evidence of diet-gut-lung interactions and associations with clinical measurements. (A) Significant correlations between dietary intake and stool bacterial populations. (B) Significant correlations between airway and stool bacterial populations. Significant correlations between stool (C) and airway (D) bacterial populations and relevant clinical outcomes in CF cohort. All bacterial taxa is identified to the genus taxonomic level. CFPE = CF pulmonary exacerbactions, FEV1% pred = predicted forced expiratory volume, BMIZ = Body mass index, controlling for age. WAZ = weight controlling for age, HAZ, height controlling for age. *P<0.05, **P<0.01, ***P<0.001
